# In Situ Absorption in Rat Intestinal Tract of Solid Dispersion of Annonaceous Acetogenins

**DOI:** 10.1155/2012/879676

**Published:** 2012-03-05

**Authors:** Yun-Jie Dang, Han-Zhou Feng, Limei Zhang, Chun-Hui Hu, Chun-Yan Zhu

**Affiliations:** Institute of Medicinal Plant Development, Chinese Academy of Medical Sciences, Beijing 100193, China

## Abstract

Isolated from *Annona squamosa L*, Annonaceous acetogenins (ACGs) exhibit a broad range of biological properties yet absorbed badly due to the low solubility. Solid dispersion in polyethylene glycol 4000 (PEG 4000) has been developed to increase the solubility and oral absorption of ACGs. The formulation of ACGS-solid dispersion was optimized by a simplex lattice experiment design and carried out by a solvent-fusion method. We studied the absorption property of ACGs in rat's intestine, which showed there was a good absorption and uptake percentages with solid dispersion. The study on uptake percentage in different regions of rat's intestine attested that the duodenum had the best permeability, followed by jejunum, ileum, and colon in order with no significant differences. So the paper drew the conclusion that solid dispersion could improve the solubility and oral absorption of annonaceous acetogenins.

## 1. Introduction

Annonaceous acetogenins (ACGs) constitute a group of natural substances that may be isolated exclusively from species of the family Annonaceae. The AGEs are one of the most interesting classes of natural products appearing in the past two decades. They exhibit a wide variety of biological activities, and, impressively, some of which have strong cytotoxic against various cancer cells. The Annonaceae is comprised of some 120 genera and include over 2100 species; most of the family is spread in tropics and subtropics. Many of the tropical species bear edible fruits and have been naturalized from central and South America to other warm areas in Asia and Africa [1–3].

ACGs are white, waxy derivatives of long-chain (C_32_ or C_34_) belonging to a series of C-35/C-37 natural products. ACGs always possess a terminal *γ*-lactone ring and a terminal aliphatic side chain connected with some oxygen-bearing moieties, such as one-to-three tetrahydrofuran (THF) and/or tetrahydropyran (THP) rings, which plays an important role in determining the relative activities of the acetogenins [4–6]. Recent studies have shown that the two functional units, the central THF system flanked by hydroxyl groups and the terminal *γ*-lactone and also the alkyl chain that acts as a spacer between them, play significant roles in inhibiting the enzyme [7, 8]. The ring systems can be single, adjacent, or nonadjacent types, and these systems, with their flanking hydroxyls, create a number of chiral centers. A complex mixture of diastereomers is the usual result.

ACGs are powerful cytotoxins and display in vivo antitumor, pesticidal, antimalarial, anthelmintic, piscicidal, antiviral, and antimicrobial properties. Commercial products include shampoo for treating infestations of head lice, fleas, and ticks, a series of pesticidal sprays, and an ointment for the treatment of oral herpes (HSV-1) and other skin afflictions [1]. As the potent inhibitors of mitochondrial (complex I) as well as cytoplasmic production of adenosine triphosphate (ATP) and related nucleotides, ACGs could induce the programmed cell death process in tumors cells at very low concentration. Although further studies of the cellular processes involved in the modulation of the selectivity of ACGs against the various tumor cells are required, AGEs have been regarded as candidates for future generations of antitumor drugs with different mechanisms.

Besides, Derbré et al. have identified new putative protein targets of squamocin, an acetogenin of the Annonaceae, ruling out the previously accepted “complex I dogma” [9].

These compounds are Complex I inhibitors, which play an important role in the maintenance of the bioenergetic function of the cell by driving the ATP synthesis from the mitochondrial-reduced equivalents produced in the central metabolic oxidative pathways [9–16].

**Figure 1:**
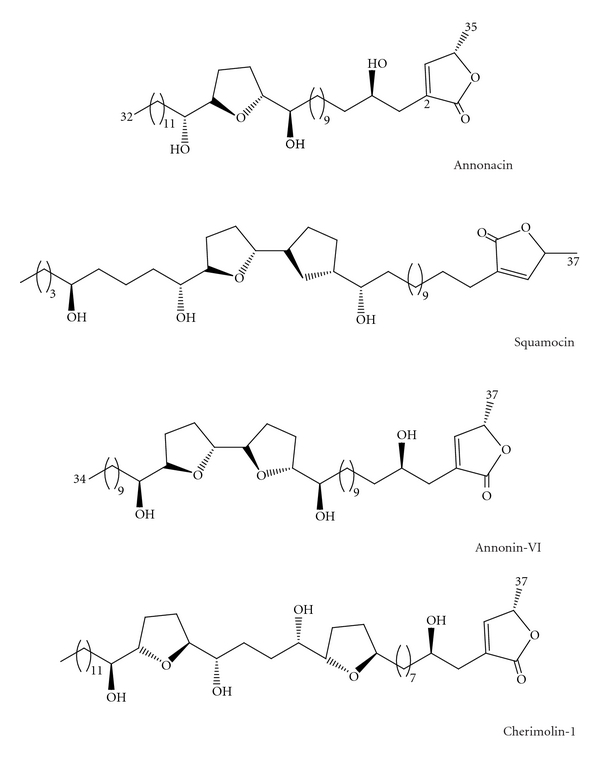
The chemical structure of ACGs compounds.

We isolated and purified several ACGs from seeds of *Annona squamosa* L. (sugar apple), such as annonacin, squamocin, annonin-VI, and cherimolin-I (Figure 1), which accounted for about 3.2%, 8%, 38%, and 9.2% in the total extracts according to the previous study in our lab.

Chermolin-I and annonacin have mono-THF, whenever squamocin and annonin-VI represent bis-THF type. The bis-THF type compounds can be organized into three major subtypes, and the squamocin and annonin-VI belonged to annonin-VI subtype [1]. Degli Esposti et al. first used mammalian mitochondria to study the action of ACGs and reported that annonin-VI inhibited the proton pumping function of complex I with similar efficiency under steady-state and nonsteady-state conditions [17]. Besides, Yuan et al. found that annonacin could arrest T24 bladder cancer cells at the G1 phase and cause cytotoxicity in a Bax- and caspase-3-related pathway [18]. Squamocin was also found to inhibit the proliferation of K562 cells via G2/M arrest in association with the induction of p21, p27 and the reduction of Cdk1 and Cdc25C kinase activities [19]. These works connected the AGEs with cell apoptosis, which exploited the multiple functions of AcGs as new anticancer candidates [20].

However, possessing various pharmacological effects and the antitumor activities in particular, ACGs were so poorly soluble as to result in being not wellabsorbed after oral administration. Therefore, the challenge of the pharmaceutical technology lies in improving the water solubility of the substances. Solid dispersions are one of the most promising strategies to improve the oral bioavailability of poorly water soluble drugs. SD is defined as the dispersion of one or more active ingredients in an inert hydrophilic carrier or matrix at solid state, prepared by the fusion, solvent, or the solvent-fusion method [21–24]. SD provides the possibility to reduce the drug particle size almost to the molecular level. Solid dispersions are also one of the most successful strategies to improve drug release of poorly soluble drugs, which can be defined as molecular mixtures of poorly water soluble drugs in hydrophilic carriers, which present a drug release profile that is driven by the polymer properties [25–28].

The present study aims to investigate the absorption of acetogenins-solid dispersion (ACGs-SD). Firstly, we prepared several SDs containing different proportions of PEG 4000 to obtain the best formulation. Then, permeability studies were carried out with the rat in situ single-pass intestinal perfusion model, as it has been shown to be predictive of in vivo absorption in humans [29–36]. We characterized the intestinal absorption of ACGs-SD by examining the concentration of chermolin-I, annonacin, squamocin and annonin-VI in rats intestinal perfusion solution. Then we evaluated the absorption sites of ACGs-SD after oral administration by a closed loop experiment.

## 2. Experimental

### 2.1. Chemicals, Reagents, and Animals

 The native compounds (ACGs) are kindly provided by natural department, Institute of Medicinal Plant Development, Chinese Academy of Medical Sciences, as well as the standard of cherimolin-I, and annonacin, squamocin, annonin-VI (purity > 98%). PEG4000 was purchased from Beijing Chemical Reagents Co. Ltd (0051230). HPLC grade methanol was purchased from Fisher Scientific Co. Ltd. (USA). Absolute alcohol was analytical reagent grade, from the BEIJING SHIJI (Beijing, China), and all other reagents were of analytical grade. Sprague-Dawley rats (SCXK (jing) 2007-0001), male, healthy, weighing 250 ± 20 g, were purchased from Vital River Laboratory Animal Technology Co. Ltd. (Beijing, China) for the animal experiment. Animals were kept in a normally controlled breeding room with standard laboratory food and water for one week prior to the experiments. The rats were maintained in accordance with internationally accepted principles for laboratory animal use and the study was approved by the Beijing Animal Care Committee.

## 3. Methods

### 3.1. Preparation of the ACGs Solid Dispersion

Solid dispersion was prepared by solvent-fusion method [22]. 5 mg ACGs and 100 mg PEG were accurately weighted and dissolved in 5 mL of alcohol, and the solvent was evaporated under 40°C and freezed quickly under −80°C for 2 h and then dried under vacuum at room temperature for 20 h. After being dried, the sample was pulverized, sieved, and the fractions ≤100 *μ*m were selected.

### 3.2. High-Performance Liquid Chromatography (HPLC)

The exact content of the compounds, cherimolin-I, annonacin, squamocin, annonin-VI, was determined using a pump L7100, and a SHIMADZU SPD-10AVP UV-detector (SHIMADAU, Japan). An SLC-10AVP (Shimadzu, Japan) equipped with a C-18 reversed-phase chromatographic column (250 × 4.6 mm; 5 *μ*m particle size) was used. The column was kept at 40°C throughout the elution process, which used a mobile phase consisting of acetonitrile and water at a total flow rate of 1.0 mL · min^−1^ and the detection wavelength set to 220 nm. The system was run with a gradient program set in Shimadzu LC solution workstation. Pump B server for acetonitrile (solvent B): 0~10 min 29%~29% B, 10~35 min 27%~27% B, 35~45 min 25%~25% B, 45~55 min 15%~15% B, 55~70 min 29%~29% B; injection volume: 20 *μ*L.

The method was fully validated for specificity, linearity, LLOQ, accuracy, and precision. Control samples (QC) were assessed by the procedure as described above to evaluate specificity of the method.

#### 3.2.1. Linearity

The calibration samples were prepared in duplicates and assayed as described above. The calibration equation was obtained by linear least-squares regression analysis with the aid of Microsoft Excel.

#### 3.2.2. Accuracy and Precision

QC samples in five replicates were analyzed on the same day to determine the intra-day precision and accuracy and on five consecutive days to determine the inter-day precision and accuracy. The concentration of each sample was determined by using the calibration standards prepared on the same day.

#### 3.2.3. Sensitivity

Sensitivity was determined by the detection limit (LLOD, three times of the value of the background noise signal, S/N = 3) and the lowest quantification limit (LLOQ, ten times of the baseline noise, lowest concentration of standard measurable).

#### 3.2.4. Stability

The stability of sample solution was also tested at room temperature and 4°C, respectively, for 24 h. For freeze-thaw stability testing, the samples were determined after three freeze (−20°C) and thaw (room temperature) cycles, and the concentration was compared to their nominal concentration. For the short-term stability, the samples (three replicates at each QC concentration) were extracted and placed at room temperature for 24 h and then injected into the HPLC system for analysis.

### 3.3. Solubility Studies

Solubility studies were conducted by placing an excess amount of ACGs (approximately 15 mg) and ACG-solid dispersion in 10 mL distilled water, and the mixture was heated at 37 ± 0.5°C in a water bath to facilitate the solubilization using a vortex mixer. Mixtures were equilibrated at 37 ± 0.5°C for 72 h in a water bath. The equilibrated samples were centrifuged at 3000 rpm for 15 min to remove the undissolved material. The supernatant was determined by HPLC.

### 3.4. Dissolution Studies

Dissolution studies were carried out according to the Chinese Pharmacopoeia 2010 apparatus No. 1 (paddle method) with a ZRS-4 dissolution apparatus (Tianjin, China). The concentration of each component is determined by HPLC. 100 mL of pH 6.8 phosphate buffer was used as dissolution medium. The dissolution tests were carried out at 37 ± 0.5°C at a rotation speed of 100 rpm. Samples of 1 mL were withdrawn at time intervals of 5, 10, 30, 60, and 120 min. The volume of dissolution medium was adjusted to100 mL by replacing each 1 mL aliquot withdrawn with 1 mL of fresh dissolution medium. The solutions were immediately filtered through 0.45 *μ*m membrane filter. Annonacin, squamocin, annonin-VI, and cherimolin-1 in ACGs and ACGs-SD were studied similarly.

### 3.5. Single-Pass Intestinal Perfusion Studies (SPIP) in Rats

We used male Sprague-Dawley rats weighing 250 ± 20 g for all perfusion studies. The drug solution was prepared by dispersing the ACGs-SD in the K-R buffer solution and installed in the cylinder, which was incubated in at 37°C water bath to maintain temperature.

The in situ SPIP experiment was conducted according to the previously published reports [29, 30]. Firstly, six male Sprague-Dawley rats were placed in respective cages and fasted overnight before the experiment with free access to water. Rats were anesthetized with an intraperitoneal injection of pentobarbital sodium (40 mg/Kg body weight, i.p. injection), restrained in a supine position, and kept at a body temperature of 37°C using infrared lamps.

Bile duct was ligated and the intestinal content was immediately washed away with 37°C saline solution at a flow rate of 5 mL/min, by cannulating polyethylene tubings (3.2 mm diameter) using a peristaltic pump (BT00-100 M, Baoding Longer Precision Pump Co., Ltd., China). The isolated segment, duodenum, jejunum, ileum, and colon were rinsed with physiological saline to clean any residual debris until the out flowing became colorless. Then, the intestinal loop was made by ligating both ends of the intestinal loop from 4 cm below the bile duct opening to a segment above the ileocecum of small intestine of anesthetized rats. Care was taken to handle the small intestine gently in order to maintain an intact blood supply. The whole surgical area was covered with a piece of sterilized gauze wetted with physiological saline solution.

ACGs-SD was dissolved in K-R buffer solution (0.35 g KCl, 1.37 g NaHCO_3_, 7.8 g NaCl, 0.22 g NaH_2_PO_4_, 0.02 g MgCl_2_, and glucose in 1.48 g/1000 mL purified water, pH 6.5) at a concentration of 200 *μ*g/mL, 300 *μ*g/mL, 500 *μ*g/mL of ACGs, and the solution was administered to the loop via the polyethylene tubing inserted into the loop. At the start of the study, the perfusion buffer containing sample solution was first perfused at a flow rate of 5 mL/min to ensure filling the segment rapidly, and the time was set to zero with the immediate start of the perfusion and the solution volume in circulation was recorded as the 0 min volume. After 10 min, when steady-state condition was achieved, the flow rate was adjusted to 0.2 mL/min, and the outlet perfused samples were collected at time intervals (5, 10, 15, 30, 60, 90, 120 min) for 2 h. During 2 h perfusion period, at each time period, assemble the outlet solution. At the end of the experiment, the length of perfused intestinal segment was measured without stretching.

### 3.6. Effects of Different Intestinal Site on Intestinal Absorption

SPIP was performed in different intestinal segments (duodenum, jejunum, ileum, and colon segment) to test if the different intestinal showed varies absorption profile [31, 32].

The study followed the method described in Li et al. [32] and Dahan and Amidon [33]. Each 10 cm long intestinal segment was isolated as follows: duodenum segment beginning from 1 cm distal to pylorus, jejunum segment beginning from 15 cm away from pylorus, ileum segment beginning at the site 20 cm upwards caecum, while segment colon beginning at the site 2 cm distal to caecum. The above segments were carefully cannulated and ligated with both the ends. Then, each segment was perfused with solution containing different mass of ACGs.

### 3.7. Stability of ACGs in K-R Buffer Solution

Exact amount of ACGs-SD was dissolved in K-R buffer solution at 37°C in water bath. Chermolin-1, annonacin, squamocin, and annonin-VI were determined at 0, 30, 60, 90, and 120 min to analysis the stability of ACGs in K-R buffer solution.

### 3.8. Data Analysis

The effective permeability (*K*_a_, *P*_app_) of the drug disappearing from the intestinal lumen during the intestinal perfusion was calculated by the steady-state equation:

(1) $$\begin{array}{c} K_{\text{a}}\;=\frac{\left(1-C_{\text{out}}Q_{\text{out}}/C_{\text{in}}Q_{\text{in}}\right)Q}{V}\\ P_{\text{app}}=\frac{\left[-\;Q_{\text{in}}\cdot \ln\;\left(C_{\text{out}}Q_{\text{out}}/C_{\text{in}}Q_{\text{in}}\right)\right]}{2\pi rL} \end{array}$$

where the radius of the gut lumen was represented by *r*, its length by *L*, and the perfusate flow rate by *Q* (mL/min). *V* presents the volume of the tested intestinal. The fraction *C*_out_/*C*_in_ represents the concentration of the dr*μ*g leaving the perfused segment (*C*_out_) normalized to the concentration entering the segment (*C*_in_). The *C*_out_ values were corrected for net water flux by the gravimetric method [34].

Once the intestinal segment reached steady state, subsequent values of *C*_out_/*C*_in_ were used to calculate *P*_app_. *P*_app_ represents the permeability of the compound (cm/s) as determined by the disappearance of the compound from the intestinal lumen. Steady state was assumed to be reached when *C*_out_/*C*_in_ reached a constant value, as determined by direct observation of the data [35].

### 3.9. Statistics

Statistical analyses were performed using SPSS13. Statistical significance was considered to be reached at *P* < 0.05.

## 4. Results and Discussion

### 4.1. Validation of HPLC Method

#### 4.1.1. Specificity

**Figure 2:**
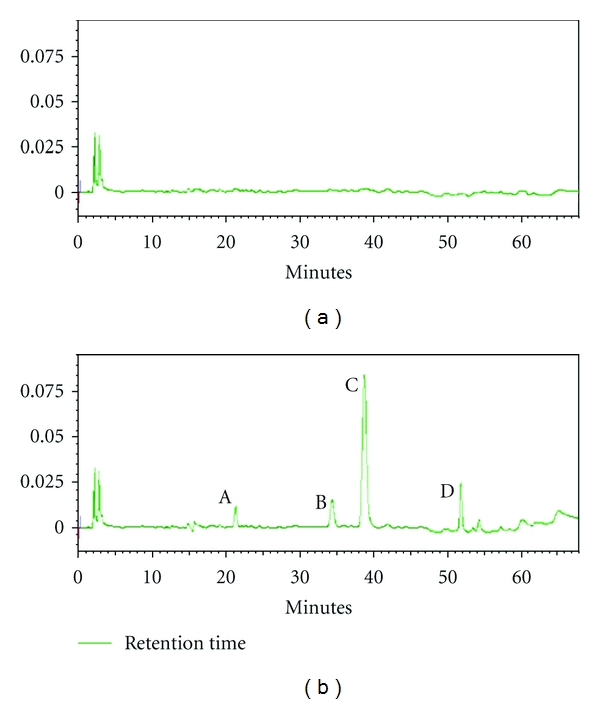
HPLC Chromatograms of A: Cherimolin-1, B: annonacin, C: Squamocin, D: annonin-VI. (a) blank K-R buffer solution; (b) the four compounds in ACGs extract.

As the Figure 2 shows, under the HPLC condition, no interference peaks were observed from the K-R buffer solution as well as the pre-dose intestinal samples from the SD rats which participated in the absorption study of ACGs. This method provided a sufficient specificity.

#### 4.1.2. Linearity

Calibration curves of ACGs were linear over the concentration range of 0.5–80 *μ*g/mL. Good linearity with a correlation coefficient *r* ^2^ = 0.999 was observed. The representative regression equation was *y* = 14514*x* − 13358 (annonacin), *y* = 16939*x* − 7166.1 (squamocin), *y* = 18691*x* + 4198.3 (annonin-VI), *y* = 16203*x* − 5353.1 (cherimolin-I), where *y* indicates the peak area of compound, and *x* indicates the concentration.

#### 4.1.3. Sensitivity

The LLOQ, defined as the lowest quantification concentration, which can be detected in plasma was 0.5 *μ*g/mL. Similarly the LLOD was 0.2 *μ*g/mL.

#### 4.1.4. Precision, Accuracy, and Recovery

This method showed good precision and accuracy. The intra-day precisions were measured to be 2.3%, 4.5%, 3.5% (annonacin), 2.2%, 3.2%, 2.7% (squamocin), 1.8%, 1.2%, 2.1% (annonin-VI), 1.8%, 1.6%, 2.6% (cherimolin-I), and the inter-day precisions were measured to be 3.2%, 2.7%, 2.1% (annonacin), 1.9%, 1.2%, 2.4% (squamocin), 1.4%, 1.8%, 2.2% (annonin-VI), 1.4%, 1.6%, 1.5% (cherimolin-I).

 The absolute recovery of ACGs from K-R buffer solution was determined to be 98.2–101.2%, the relative recovery of ACGs was determined to be 98.4–105.1%.

#### 4.1.5. Stability

Stock solutions of ACGs were stable for at least 30 days when stored at 4°C. No significant change was found after three freeze-thaw cycles and stored at room temperature for 24 h (Table 1).

### 4.2. Solubility Studies

The solubility of free AGCs is so poor that the concentration is out of the lowest quantification limit of the analysis method, whereas the solid dispersion technology increased the solubility of AGCs remarkably. The solubility of annonacin is 213.11 *μ*g/mL, squamocin is 1084.73 *μ*g/mL, annonin-VI is 249.54 *μ*g/mL, and cherimolin-I is 99.33 *μ*g/mL. Solid dispersion could reduce the drug particle size almost to the molecular to enhance the solubility of drug. The data displayed that solid dispersion could increase ACGs solubility significantly.

### 4.3. In Vitro Dissolution Studies

**Figure 3:**
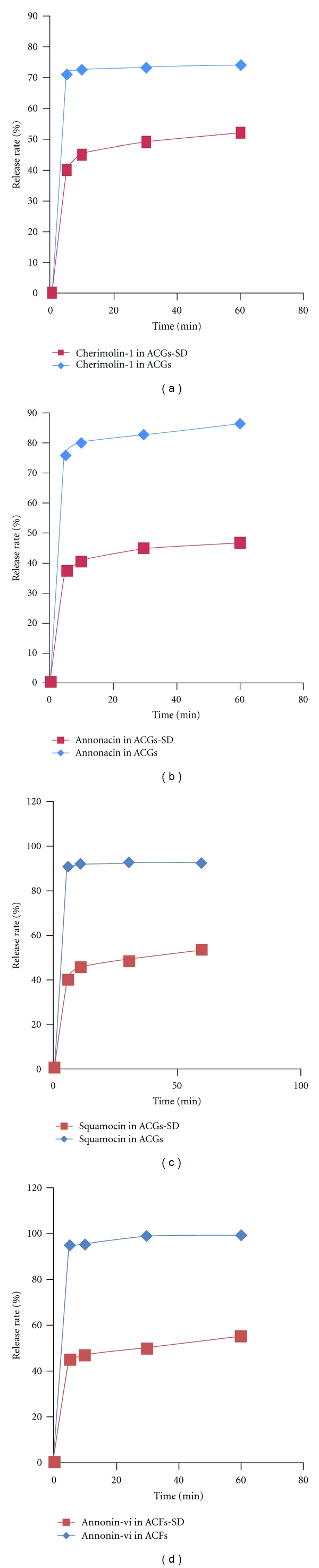
Dissolution profiles of ACGs and ACGs-SD in PBS (pH 6.8) at 37°C.

It is important to improve the drug solubility in the gastrointestinal tract to increase the oral adsorption of poorly water soluble drugs. The four compounds in ACGs-SD were stable in K-R buffer solution for 2 h (Table 2) and tested for dissolution properties, the results shown in Figure 3. At each time point, the chemical dissolved from the solid dispersion state was significantly higher than that of free ACGs. This profile could ascribe to the solubilizing effect of PEG4000 and the high dispersion state.

### 4.4. Dose Dependency in In Situ Intestinal Absorption of ACGs

**Figure 4:**
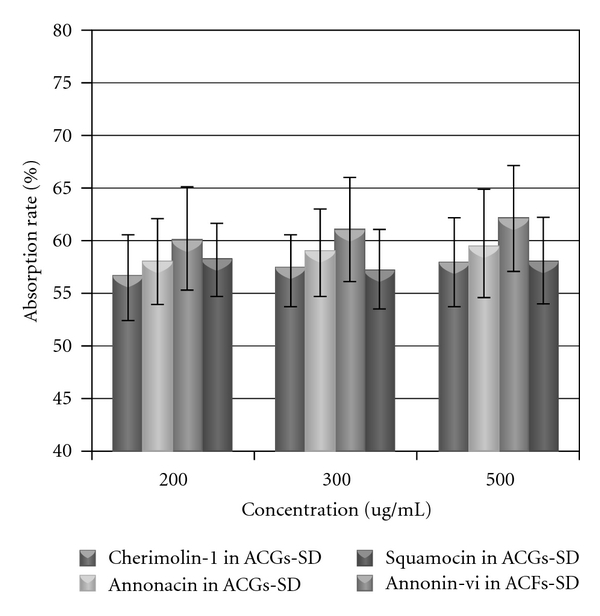
Intestinal absorption of annonacin, squamocin, annonin-VI and Cherimolin-1 in rats. ACGs-SD was administered at a dose of 200, 300, 500 *μ*g/mL to a 10 cm-long jejunum loop and the intestinal absorption rate (%) was estimated by measuring the remained amount of ACGs in the loop 2 h after administration. (*n* = 5).

The dose dependency in the intestinal absorption of ACGs was examined by administering at a dose of 200 *μ*g/mL, 300 *μ*g/mL, 500 *μ*g/mL (total extract) to the intestinal loop in rats separately (Table 3). The absorption percentage of ACGs was estimated by the disappearance percentage from the loop for 2 h, and there is no significantly difference among the three-dose group (Figure 4).

### 4.5. Effects of Different Intestinal Site on Intestinal Absorption

The effect of different intestinal site on intestinal absorption of ACGs was examined by administering at a dose of 300 *μ*g/mL to the different intestinal loop in rats (Table 4). The absorption percentage of ACGs was comparable among the four regions (duodenum, jejunum, ileum, and colon segment). The results showed that there is no significant difference among different intestinal site.

## 5. Discussion

Oral drug delivery is the simplest and easiest way of administering drugs. In fact, most drugs are poorly water soluble drugs, not well absorbed after oral administration. Comparing with most plant constituents with low oral bioavailability, ACGs exhibit a broad range of biological properties, but hardly soluble in water. Drug release is a crucial and limiting step for oral drug bioavailability, particularly for drugs with low gastrointestinal solubility and high permeability. By improving the drug release profile of these drugs, it is possible to enhance their bioavailability and reduce side effects [36, 37].

In the present study, we prepared ACGs-SD with PEG4000 then evaluated the absorption sites of ACGs-SD by administering into the intestinal loop prepared at different sites along the small intestine and dose dependence in in situ intestinal absorption. Such study on the intestinal absorption sites would be expected to prove the adsorption improvement effect of solid dispersion and reveal the best absorption site in intestinal. The absorption data showed that ACGs-SD displayed good absorption in intestinal, the percentage of absorption all above 50%. Although the uptake study of different site of intestinal showed duodenum had the best permeability, followed by jejunum, ileum, and colon, there is no significant difference among the four intestinal sites.

Solid dispersions are one of the most successful strategies to improve drug release of poorly soluble drugs. These can be defined as molecular mixtures of poor water soluble drugs in hydrophilic carriers, which present a drug release profile that is driven by the polymer properties [38].

## 6. Conclusion

This study clearly revealed that the preparation of solid dispersions of PEG 4000 with ACGs led to enhanced solubility and bioavailability. The ACGs existed in an amorphous state. The comparison of dissolution of ACGs and its solid dispersion with PEG 4000 indicated that the solid dispersion had a higher release profile. The single-pass intestinal perfusion studies (SPIPs) in rats also confirmed that ACGs-SD displayed a good absorption in intestinal. In conclusion, our results demonstrated that formulation of ACGs with PEG significantly improved the four compounds' solubility and dissolution properties; the resulting product also could improve the absorption of ACGs in intestinal.

## Figures and Tables

**Table 1:** Stability test of ACGs during the storing and preparation procedures.

Nominal concentration (ng/mL)	Accuracy (mean ± SD)%	
	Freeze-thaw (three cycles)	Samples at room temperature for 24 h
Annonacin	96.0 ± 1.0	96.3 ± 2.1
Squamocin	101.6 ± 4.2	101.3 ± 6.2
Annonin-VI	99.3 ± 5.0	98.3 ± 2.1
Cherimolin-I	99.5 ± 1.2	99.1 ± 2.1

**Table 2:** Stability of ACGs in K-R buffer solution (*n* = 3).

Sample	Time (h)				RSD (%)
	0	0.5	1	2	
Annonacin	100%	98.63%	98.59%	96.16%	1.62
Squamocin	100%	100.51%	99.86%	98.58%	0.82
Annonin-VI	100%	95.64%	99.59%	85.83%	2.40
Cherimolin-I	100%	100.56%	99.58%	99.74%	0.42

**Table 3:** The permeability (*P*_app_, Mean ± SD, *n* = 6) at different perfusate concentration.

Concentration (*μ*g · mL^−1^)	*P*_app_ × 10^3^ (cm · s^−1^) ± SD			
	Annonacin	Squamocin	Annonin-VI	Cherimolin-I
200	1.29 ± 0.12	0.96 ± 0.28	0.75 ± 0.37	1.18 ± 0.23
300	0.92 ± 0.23	0.96 ± 0.27	0.75 ± 0.21	0.79 ± 0.38
500	0.69 ± 0.31	0.93 ± 0.32	0.79 ± 0.30	0.75 ± 0.29

**Table 4:** The permeability (*P*_app_, Mean ± SD, *n* = 6) at different site of intestinal.

Intestine segment	*P*_app_ × 10^3^ (cm · s^−1^) ± SD			
	Annonacin	Squamocin	Annonin-VI	Cherimolin-I
Duodenum	1.36 ± 0.34	1.24 ± 0.27	2.28 ± 0.29	1.21 ± 0.58
Jejunum	1.38 ± 0.82	1.30 ± 0.27	1.87 ± 0.29	1.18 ± 0.52
Ileum	1.17 ± 0.49	1.14 ± 0.31	1.26 ± 0.30	2.25 ± 0.4
colon	0.95 ± 0.45	1.03 ± 0.19	1.10 ± 0.45	1.59 ± 0.31

## References

[1] Alali FQ, Liu XX, McLaughlin JL (1999). Annonaceous acetogenins: recent progress. *Journal of Natural Products*.

[2] Bermejo A, Figadère B, Zafra-Polo MC, Barrachina I, Estornell E, Cortes D (2005). Acetogenins from annonaceae: recent progress in isolation, synthesis and mechanisms of action. *Natural Product Reports*.

[3] McLaughlin JL (2008). Paw paw and cancer: annonaceous acetogenins from discovery to commercial products. *Journal of Natural Products*.

[4] Ye Q, He K, Oberlies NH (1996). Longimicins A-D: novel bioactive acetogenins from Asimina longifolia (annonaceae) and structure-activity relationships of asimicin type of annonaceous acetogenins. *Journal of Medicinal Chemistry*.

[5] Oberlies NH, Chang CJ, McLaughlin JL (1997). Structure-activity relationships of diverse annonaceous acetogenins against multidrug resistant human mammary adenocarcinoma (MCF-7/Adr) cells. *Journal of Medicinal Chemistry*.

[6] Yang S, Yu J, Xu L (2000). Chemical constituents of annonaceae plants and their antitumor activities. *Acta Academiae Medicinae Sinicae*.

[7] Tormo JR, Gallardo T, Aragón R, Cortes D, Estornell E (1999). Specific interactions of monotetrahydrofuranic annonaceous acetogenins as inhibitors of mitochondrial complex I. *Chemico-Biological Interactions*.

[8] Gallardo T, Saez J, Granados H (1998). 10-oximeguanacone, the first nitrogenated acetogenin derivative found to be a potent inhibitor of mitochondrial complex I. *Journal of Natural Products*.

[9] Derbré S, Gil S, Taverna M (2008). Highly cytotoxic and neurotoxic acetogenins of the Annonaceae: new putative biological targets of squamocin detected by activity-based protein profiling. *Bioorganic and Medicinal Chemistry Letters*.

[10] Cavé A, Figadère B, Laurens A, Cortes D (1997). Acetogenins from Annonaceae. *Fortschritte der Chemie organischer Naturstoffe*.

[11] Zafra-Polo MC, Figadère B, Gallardo T, Tormo JR, Cortes D (1998). Natural acetogenins from annonaceae, synthesis and mechanisms of action. *Phytochemistry*.

[12] Zeng L, Ye Q, Oberlies NH (1996). Recent advances in annonaceous acetogenins. *Natural Product Reports*.

[13] Kuwabara K, Takada M, Iwata J (2000). Design syntheses and mitochondrial complex I inhibitory activity of novel acetogenin mimics. *European Journal of Biochemistry*.

[14] Lannuzel A, Michel PP, Höglinger GU (2003). The mitochondrial complex I inhibitor annonacin is toxic to mesencephalic dopaminergic neurons by impairment of energy metabolism. *Neuroscience*.

[15] Escobar-Khondiker M, Höllerhage M, Muriel MP (2007). Annonacin, a natural mitochondrial complex I inhibitor, causes tau pathology in cultured neurons. *Journal of Neuroscience*.

[16] Yuan SSF, Chang HL, Chen HW (2006). Selective cytotoxicity of squamocin on T24 bladder cancer cells at the S-phase via a Bax-, Bad-, and caspase-3-related pathways. *Life Sciences*.

[17] Degli Esposti M, Ghelli A, Ratta M, Cortes D, Estornell E (1994). Natural substances (acetogenins) from the family Annonaceae are powerful inhibitors of mitochondrial NADH dehydrogenase (Complex I). *Biochemical Journal*.

[18] Yuan SSF, Chang HL, Chen HW (2006). Selective cytotoxicity of squamocin on T24 bladder cancer cells at the S-phase via a Bax-, Bad-, and caspase-3-related pathways. *Life Sciences*.

[19] Lu MC, Yang SH, Hwang SL (2006). Induction of G2/M phase arrest by squamocin in chronic myeloid leukemia (K562) cells. *Life Sciences*.

[20] Liaw CC, Wu TY, Chang FR, Wu YC (2010). Historic perspectives on Annonaceous acetogenins from the chemical bench to preclinical trials. *Planta Medica*.

[21] Chiou WL, Riegelman S (1971). Pharmaceutical applications of solid dispersion systems. *Journal of Pharmaceutical Sciences*.

[22] Ford JL (1986). The current status of solid dispersions. *Pharmaceutica Acta Helvetiae*.

[23] Leuner C, Dressman J (2000). Improving drug solubility for oral delivery using solid dispersions. *European Journal of Pharmaceutics and Biopharmaceutics*.

[24] Sekiguchi K, Obi N (1961). Studies on absorption of eutectic mixtures. I. A comparison of the behaviour of eutectic mixture of sulfathiazole and that of ordinary sulfathiazole in man. *Chemical & Pharmaceutical Bulletin*.

[25] Moneghini M, Kikic I, Voinovich D, Perissutti B, Filipović-Grčić J (2001). Processing of carbamazepine—PEG 4000 solid dispersions with supercritical carbon dioxide: preparation, characterisation, and in vitro dissolution. *International Journal of Pharmaceutics*.

[26] Leuner C, Dressman J (2000). Improving drug solubility for oral delivery using solid dispersions. *European Journal of Pharmaceutics and Biopharmaceutics*.

[27] Okonogi S, Oguchi T, Yonemochi E, Puttipipatkhachorn S, Yamamoto K (1997). Improved dissolution of ofloxacin via solid dispersion. *International Journal of Pharmaceutics*.

[28] Vasconcelos T, Sarmento B, Costa P (2007). Solid dispersions as strategy to improve oral bioavailability of poor water soluble drugs. *Drug Discovery Today*.

[29] Lennernäs H (1998). Human intestinal permeability. *Journal of Pharmaceutical Sciences*.

[30] Mori N, Yokooji T, Kamio Y, Murakami T (2008). Characterization of intestinal absorption of mizoribine mediated by concentrative nucleoside transporters in rats. *European Journal of Pharmacology*.

[31] Kim JS, Mitchell S, Kijek P, Tsume Y, Hilfinger J, Amidon GL (2006). The suitability of an in situ perfusion model for permeability determinations: utility for BCS class I biowaiver requests. *Molecular Pharmaceutics*.

[32] Li H, Zhao X, Ma Y, Zhai G, Li L, Lou H (2009). Enhancement of gastrointestinal absorption of quercetin by solid lipid nanoparticles. *Journal of Controlled Release*.

[33] Dahan A, Amidon GL (2009). Segmental dependent transport of low permeability compounds along the small intestine due to P-glycoprotein: the role of efflux transport in the oral absorption of BCS class III drugs. *Molecular Pharmaceutics*.

[34] Soria I, Zimmerman CL (1994). Intestinal absorption of (-)-carbovir in the rat. *Pharmaceutical Research*.

[35] Eriksson AH, Varma MVS, Perkins EJ, Zimmerman CL (2010). The intestinal absorption of a prodrug of the mGlu2/3 receptor agonist LY354740 is mediated by PEPT1: in situ rat intestinal perfusion studies. *Journal of Pharmaceutical Sciences*.

[36] Streubel A, Siepmann J, Bodmeier R (2006). Drug delivery to the upper small intestine window using gastroretentive technologies. *Current Opinion in Pharmacology*.

[37] Tanaka N, Imai K, Okimoto K (2006). Development of novel sustained-release system, disintegration-controlled matrix tablet (DCMT) with solid dispersion granules of nilvadipine (II): in vivo evaluation. *Journal of Controlled Release*.

[38] Vasconcelos T, Sarmento B, Costa P (2007). Solid dispersions as strategy to improve oral bioavailability of poor water soluble drugs. *Drug Discovery Today*.

